# Role of Immunohistochemistry in the Differential Diagnosis of Pediatric Renal Tumors: Expression of Cyclin D1, Beta-Catenin , PDGFR-Alpha, and PTEN

**DOI:** 10.5146/tjpath.2022.01568

**Published:** 2022-05-19

**Authors:** Nuray Kepil, Şebnem Batur, Zeynep Ecem Kain, Gamze Özcan, Şenol Emre, Rahşan Özcan, Tülin Tiraje Celkan, Nil Çomunoğlu

**Affiliations:** Department of Pathology, Istanbul University, Cerrahpasa Faculty of Medicine, Istanbul, Turkey; Department of Pediatric Surgery, Istanbul University, Cerrahpasa Faculty of Medicine, Istanbul, Turkey; Department of Pediatrics Oncology, Liv Hospital, Istanbul, Turkey

**Keywords:** Cyclin D1, Immunohistochemistry, Pediatric pathology, Renal tumors, Wilms tumor

## Abstract

*
Objective: *Pediatric renal tumors overlap histomorphologically and may cause misdiagnosis. We aimed to determine the role of immunohistochemical staining of Cyclin D1, PTEN, beta-catenin and PDGFR-alpha on pediatric renal tumors.

*
Material and Method: *Thirty-six cases of 8 different tumors were included in the study. Four blocks of paraffin tissue microarray were constructed. Cyclin D1, PTEN, beta-catenin and PDGFR-alpha were used in all cases. Staining intensity and extent were graded.

*
Results: *All cases of clear cell sarcoma (CCS) and epithelial components of Wilms tumor (WT) showed immunopositivity for Cyclin D1 but blastemal and stromal components of WT were negative. All cases of CCS and most cases of WT consisting of blastemal and stromal components demonstrated loss of expression with PTEN.

*
Conclusion: *Cyclin D1 is not a specific immunohistochemical marker due to its strong and diffuse positivity in CCS cases. It may be useful to differentiate CCS from blastemal and stromal components of WT. Other markers except cyclin D1 do not have a role in the differential diagnosis.

## INTRODUCTION

Wilms tumor (WT) is the most common genitourinary tumor of the children aged between 2-4 years. Having triphasic components, which are blastemal, epithelial, and stromal, it should be taken into the differential diagnosis with other renal tumors. Clear cell sarcoma (CCS) is one of the mesenchymal tumors of kidney, which is frequently seen in the third year of life. Histologically, epithelioid cells with round to oval nucleus form nests and cords. Malignant rhabdoid tumor of kidney is a prominently aggressive tumor, seen among children under 10 years. It is composed of rhabdoid cells that have eosinophilic nucleolus and cytoplasm, big round nucleus, and paranuclear inclusions. These cells are epithelioid, round, and polygonal in appearance and show a solid and trabecular growth pattern (1). Mesoblastic nephroma, classified into two groups as classical and cellular, is one of the mesenchymal tumors with low potential of malignancy and is seen among children younger than 3 years. Histologically, tumor cells form fascicles composed of spindle cells and may resemble infantile fibromatosis.

Although clinical and radiological findings may be helpful in differential diagnosis, all of the morphological findings of renal pediatric tumors may overlap with the various subtypes of Wilms tumor (2,3). Bi- or triphasic Wilms tumor diagnosis might be mistaken with other pediatric renal tumors in tru-cut biopsy materials as well as monophasic Wilms tumor in nephrectomy materials. Wilms tumor which has only pure blastemal component may be confused with Ewing sarcoma and neuroblastoma; components showing rhabdoid differentiation may be confused with malignant rhabdoid tumor; and the stromal component may be confused with clear cell sarcoma and mesonephric nephroma. The stroma of Wilms tumor may be in similar appearance to renal clear cell sarcoma (CCS), particularly after pre-operative chemotherapy (3,4).

Cyclin D1, PTEN, Beta-catenin, and PDGF-alpha are the pathways that play a role in pathogenesis of these tumors mentioned above. Thus, the immunohistochemical work-up may help to differentiate tumors with a similar appearance. Recently, the YWHAE-FAM22 rearrangement, which is shown in high grade endometrial stromal sarcoma, was reported in CCS cases and this rearrangement was resulted in upregulation of Cyclin D1. This immunohistochemical marker is recommended for differential diagnosis of tumors resembling CCSs (3,5,6).

MicroRNA is present in various biological processes such as growth, development, and metabolism. It was shown that microRNA has a substantial role in the pathogenesis of renal diseases. Above all, its solid role in the progression of Wilms tumor was demonstrated. Some studies depicted that dysregulation of microRNAs starts activation of phosphatase and tensin homologue (PTEN) / phosphoinositide 3-kinase (PI3K)/protein kinase B (Akt) signaling pathway. It was shown that this pathway plays a role in the pathogenesis of Wilms tumor; PTEN positivity positively correlated with the clinical stage and negatively correlated with metastasis to lymph nodes (7,8).

The signalling pathway of WNT/beta-catenin has a role in processes such as embryonic growth, tumorogenesis, cell proliferation, differentiation, migration, and apoptosis. Wilms tumor’s protein is a transcription factor that is negatively correlated with the WNT/beta-catenin pathway. Several studies have shown that the signaling pathway of WNT/beta-catenin is activated in Wilms tumor (9,10).

PDGF is an angiogenic factor which is formed of PDGF-A and B chains and is coded by 2 different genes. PDGF was produced in the normal kidney and Wilms tumor cells in vitro. Studies on Wilms tumor have indicated that the PDGF-A and PDGF-alpha receptors are expressed in the epithelial component (11).

In our study, we evaluated the staining features of immunohistochemical markers, such as Cyclin D1, PTEN, Beta-catenin, PDGFR-alfa, in morphologically overlapping tumors and compared our results with recent articles.

## MATERIALS and METHODS

The surgical pathology database of the Department of Pathology, Istanbul University - Cerrahpasa Faculty of Medicine was searched for pediatric renal tumors between the years of 2000 and 2018. A total of 36 cases of 16 WT (all cases were post-chemotherapy resections), 10 CCS, 3 cellular mesoblastic nephroma (CeMN), 2 classical mesoblastic nephroma (CMN), 2 malignant rhabdoid tumor, one Ewing sarcoma, one diffuse large B-cell lymphoma (DLBCL), and one malignant solitary fibrous tumor (MSFT), were included in the study. All cases were diagnosed by one pediatric and one renal pathologist. Verbal informed consent was obtained from the patients.

All tissues were fixed in 10% formalin and embedded in paraffin. Four tumor tissue microarrays (TMA) blocks were constructed, containing representative 4-micron thick sections, and processed as previously described (12). Each component of Wilms tumor was sampled on TMA blocks. Deparaffinization was performed using solutions and they were rehydrated using a series of decreasing alcohol concentrations. Samples were kept in 10 mmol/L buffered citrate solution for 30 minutes at 36 °C. PTEN (Roche, SP218), Beta-catenin (Roche, 14), PDFGR-alpha (Thermo Scientific, Ab-1), Cyclin D1 (Roche, SP4-R) immunohistochemical markers were employed with an automatic device (BenchMark XT IHK/ISH Staining Module, Ventana Medical Systems Ins., Medical Systems, Tucson, AZ, USA), according to the manufacturer’s instructions. Staining intensity was graded as weak (+), moderate (++), or strong (+++) whereas the extent of staining was graded according to the percentage. Non-staining and weak staining below 5% were considered to be negative.

### Data Analysis

All data have been presented as mean or median or in numbers and percentages. Statistical comparisons and tests for survival analyses were not performed due to the low number of subjects.

## RESULTS

The mean age of the 10 CCS cases was 12.5 (1-53 years) and the female/male ratio was 3/7. The mean age of the 16 WT cases was 5.18 (1-15 years) and the female/male ratio was 9/7. The mean age of the mesoblastic nephroma cases was 19 months (9 months - 3 years) and the female/male ratio was 2/1. The mean age of the classical/congenital mesoblastic nephroma cases was 2 and both patients were male. The mean age of the 2 malignant rhabdoid tumor cases was 18 months (12 months - 2 years) and the female/male ratio was 1/1. The Ewing sarcoma patient was female and 36 years old. The diffuse large B-cell lymphoma patient was female and 8 years old. The malignant solitary fibrous tumor patient was male and 4 years old. The immunohistemical staining features are summarized in theTable 1.

**Table 1:** Immunohistochemical staining features of renal tumors.

	**Cyclin D1**			**Beta-catenin**			**PTEN**			**PDGFR-alpha**			**Total number**
	**Positive**	**Negative**	**Extent (%)**	**Positive**	**Negative**	**Extent (%)**	**Positive**	**Negative**	**Extent (%)**	**Positive**	**Negative**	**Extent (%)**	
**CCS**	10	0	10-90	7	3	30-80	0	10	-	9	1	20-100	10
**WT-Epithelial**	7	4	10-20	11	0	40-80	11	0	100	9	-	40-80	11
**WT-Blastemal**	0	14	-	13	1	10-80	13	1	100	12	2	30-80	14
**WT-Stromal**	0	16	-	12	4	10-80	15	1	100	14	2	20-80	16
**CeMN**	2	1	40	3	0	60-80	3	0	100	3	0	50-90	3
**CMN**	0	2	-	0	2	-	0	2	-	0		-	2
**RT**	0	2	-	2	0	10-60	2	0	100	2	0	10-60	2
**EW**	0	1	-	0	1	-	0	1	-	0	1	-	1
**SFT**	0	1	-	1	0	90	1	0	100	1	0	80	1
**DLBCL**	0	1	-	0	1	-	1	0	100	1	0	90	1

**CeMN:** Cellular mesoblastic nephroma, **CMN: **Classical mesoblastic nephroma, **RT: **Rhabdoid tumor,** EW:** Ewing sarcoma, **SFT: **Solitary fibrous tumor, **DLBCL:** Diffuse large B cell lymphoma.

### Cyclin D-1

**Figure 1:**
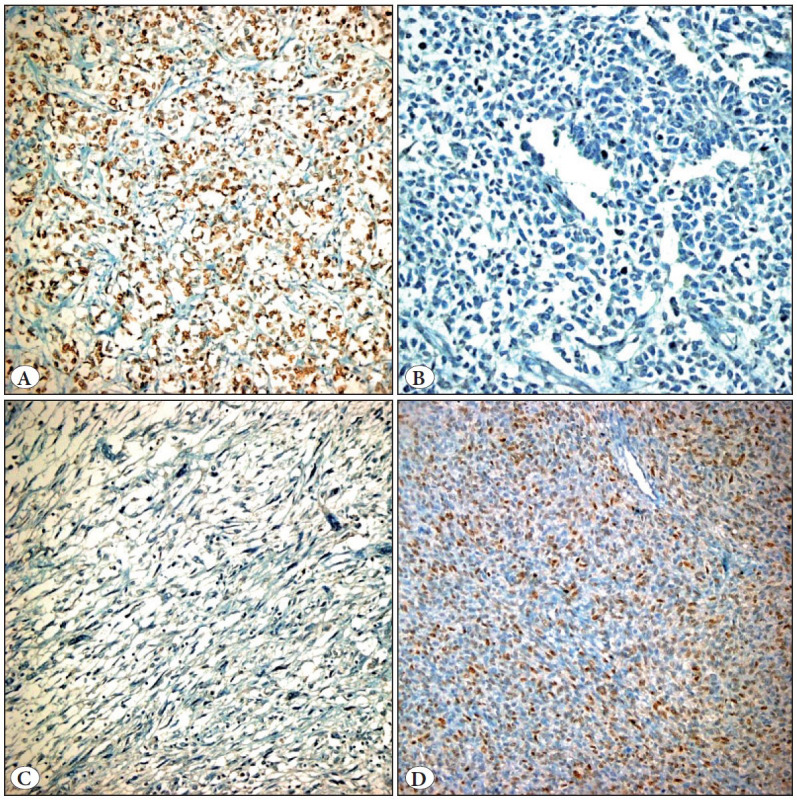
Cyclin D1 staining features.**A)**Clear cell sarcoma (x200),**B)**WT-Blastemal component (x400),**C)**WT-Stromal component (x200),**D)**Cellular mesoblastic nephroma (x200).

All 10 CCS cases stained with Cyclin D-1. Staining extent varied between 10-90% (Figure 1A). The intensity of staining was weak (+) to strong (+++). Seven out of 11 WT cases containing an epithelial component stained moderately with an extent of 10-20%. Fourteen cases that had a blastemal component (Figure 1B) and all 16 WT cases containing a stromal component (Figure 1C) showed immunonegativity. Two of the 3 CeMN cases were not stained, and the remaining one showed moderate staining with an extent of 40% (Figure 1D). This case was re-evaluated on H&E slides. Morphological features of the cells and the expansive growth pattern led us to consider it as clear cell sarcoma.

Two CMN and two malignant rhabdoid tumor cases showed immunonegativity. One Ewing sarcoma, one MSFT, and the DLBCL cases were not stained.

### Beta-Catenin

**Figure 2:**
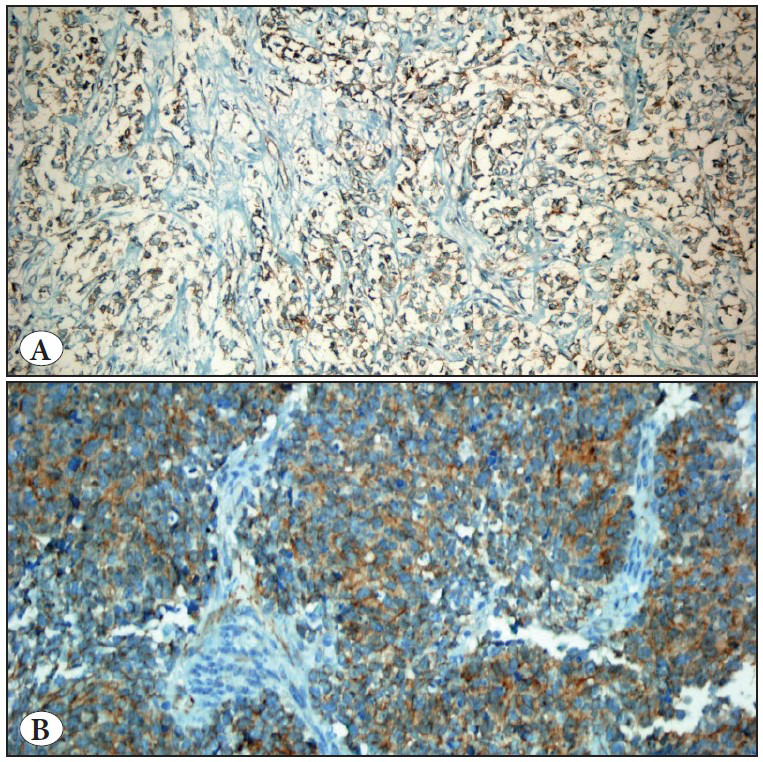
Beta-catenin staining features.**A)**clear cell sarcoma (x200),**B)**WT-Blastemal component (x400).

A cytoplasmic staining pattern was considered as positive. Three of the 10 CCS cases were negative, and 7 cases showed weak to moderate staining with a extent of 30-80% (Figure 2A). Among the 16 WT cases, all 11 cases consisting of an epithelial component showed cytoplasmic, weak to moderate immunopositivity with an extent of 40-80%. One of the 14 cases with a blastemal component was negative and the remaining cases showed weak-moderate staining with 10-80% extent (Figure 2B). Out of the 16 Wilms tumor containing a stromal component, one case showing rhabdoid features stained strongly with an extent of 80%. Four of the cases were negative. The remaining cases showed weak to moderate staining with 10-80% extent. One Ewing sarcoma and one DLBCL were negative. Two CMN cases were negative. Three CeMN cases showed moderate staining with 60-80% extent. Two malignant rhabdoid tumor cases showed weak to moderate staining with an extent of 10-60%. One MSFT case showed strong staining with 90% extent.

### PTEN

All 10 CCS cases were negative with PTEN. Among 16 Wilms tumors, 11 cases that had an epithelial component showed weak staining with 100% extent, one case containing blastemal component was negative, and the remaining cases showed weak staining with an extent of 100%. One of the 16 cases that contained a stromal component was negative and 5 showed moderate staining with 30-100% extent; two of these 5 cases were composed of rhabdoid areas. The WT case showing negativity in the blastemal component was Stage 3, and the WT case showing negativity in stromal component was Stage 2. Other cases stained weakly with an extend of 100%. One of the two cases showing anaplasia in stromal cells had weak staining with 100% extent and the other was negative.

All three CeMN cases showed weak positivity with an extent of 100%. Two congenital/classical type mesoblastic nephroma cases were negative. Two malignant rhabdoid tumor and one MSFT case stained weakly with 100% extent. One Ewing sarcoma was negative. One DLBCL showed moderate staining with an extent of 100%.

### PDGFR-Alpha

**Figure 3:**
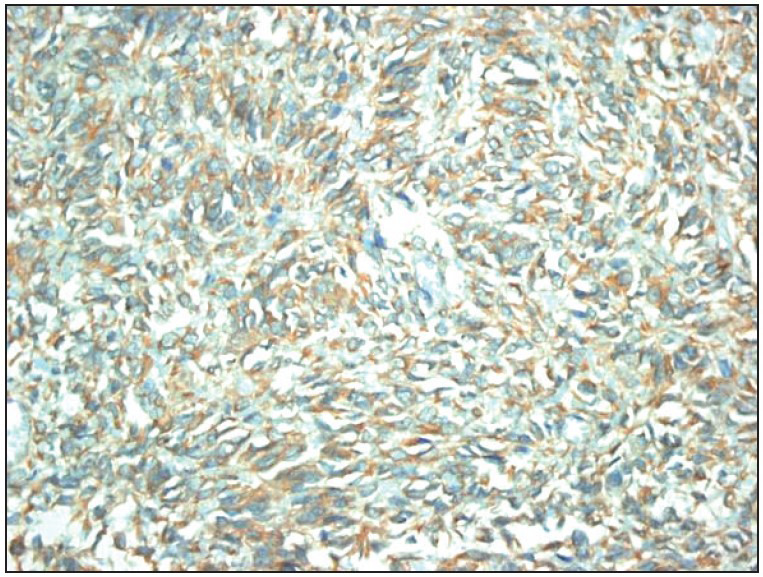
PDGFR-alpha positivity of CCS (x400).

One of the 10 CCS cases was negative, and 9 cases showed weak to moderate staining with an extent of 20-100% (Figure 3). In two out of 11 cases with epithelial components, the PDGFR stain could not be evaluated due to technical reasons. The remaining 9 cases showed weak to moderate staining with 40-80% extent. Twelve cases with a blastemal component stained weak to moderate, with an extent of 30-80%. Two cases with rhabdoid features were negative. In fourteen cases containing stromal components, weak staining was observed with an extent of 20-80%.

Three CeMN cases showed weak to moderate staining with an extent of 50-90%. One Ewing sarcoma and 2 CMN cases were negative. Two malignant rhabdoid tumor cases showed weak to moderate staining with 10-60% extent. One case of MSFT showed moderate staining with an extent of 80%. One case of DLBCL showed moderate staining with an extent of 90%.

## DISCUSSION

Wilms tumor, clear cell sarcoma, atypical teratoid rhabdoid tumor, and mesoblastic nephroma are pediatric renal tumors and less frequently Ewing sarcoma has been reported in this localization. We investigated the role of these immunohistochemical markers in the differential diagnosis:

### Cyclin D-1

Although there are studies suggesting that immunohistochemical markers can be helpful in these tumors, immunohistochemistry is limited in the differential diagnosis (2,3). Cyclin D-1 as an immunohistochemical marker that has been studied in pediatric renal tumors and has recently been proposed as a sensitive marker for clear cell sarcomas (3,5,6,13). Jet Aw et al., Mirkovic et al., and Uddin et al. reported immunopositivity in their CCS series with 8, 14, and 19 cases respectively (3,6,13). In our study, immunopositivity with cyclin D-1 was observed in all 10 CCS cases.

In the study of Jet Aw et al.*,* cyclin D-1 was immunonegative in the blastemal and stromal components of 8 Wilms tumors, whereas the epithelial components showed immunopositivity (13). Mirkovic et al. reported focal positivity in the blastemal component in 18 out of 20 WT cases. The epithelial component showed immunopositivity in most of these cases. Uddin et al. reported that one of the 9 WT cases showed weak positivity in the blastemal component and 7 were positive in the epithelial component (3). In our study, staining intensity was weak to strong with 10-90% of extent in the epithelial components. Blastemal and stromal components were negative.

The dysregulated genes of the G1-S phase of the cell cycle in Wilms tumor have been reported previously. This finding explains the cyclin D-1 immunexpression in the epithelial component of Wilms tumors (14). Staining of blastemal and stromal component was not observed in the Wilms tumor in most studies, that is compatible with ours. Cyclin D1 may be recommended as an immunohistochemical marker in the differential diagnosis of CCS and Wilms tumor.

Morphologically, the classic mesoblastic nephroma con-sists of a uniform, fibromatosis-like proliferation of fusiform cells with a fascicular appearance and it might be confused with the stromal component of Wilms tumor, and CCS. Jet Aw et al*.,* Mirkovic et al., and Uddin et al. have reported Cyclin D-1 positivity in classical mesonephric blastoma cases (3,6,13). In our study, 2 out of 5 mesoblastic nephroma cases were classical and 3 of them were cellular. Only one of these CeMN cases showed diffuse nuclear positivity with Cyclin D-1. Cyclin D-1 is not a helpful immunohistochemical marker in the differential diagnosis of CCS with mesoblastic nephromas as there are varying rates of positivity and negativity reported.

Jet Aw et al. reported patchy immunopositivity in their 6-case series, Mirkovic et al. reported focal positivity in 4 rhabdoid tumor cases, and Uddin et al. reported moderate staining in 3 of their 4 cases (3,6,13). In our study, cyclin D-1 was negative in 2 malignant rhabdoid tumors. Due to the variable staining characteristics of Cyclin D1 in malignant rhabdoid tumors, it cannot be recommended as an immunohistochemical marker in the differential diagnosis.

While cyclin D-1 showed diffuse and strong immunoposi-tivity in 3 of 5 Ewing sarcoma cases of Mirkovic et al. and 3 of 4 cases of Uddin et al., one Ewing sarcoma was immunonegative in our study (3,6). Since the number of cases was limited and different staining characteristics were reported in the literature, the role of Cyclin D-1 in the differential diagnosis of Ewing sarcoma from other tumors was not fully determined.

Studies have reported diffuse and strong staining with cyclin D-1 in neuroblastoma cases (3,6,13). Neuroblastoma cases were not included in our study because we could not find any neuroblastoma cases located at the kidney in our archive. However, in our study, negative staining with Cyclin D-1 was detected in a malignant solitary fibrous tumor and diffuse large B-cell lymphoma cases, which are very rare in the kidney.

Cyclin D-1 is a useful immunohistochemical marker due to its strong and diffuse positivity in renal CCS cases and it might be used to differentiate CCS from the blastemal and stromal component of Wilms tumor.

### Beta-Catenin

The catenin beta-1 (CTNNB1) gene encodes the protein of beta-catenin and the mutation of this gene primarily affects the WNT-signaling pathway. As a result, the protein of beta-catenin is stabilized, and its transcription is increased. The pathway of the aberrant WNT/beta-catenin leads to developmental malformations and associated malignancies. The pathway of WNT/beta-catenin is frequently activated in Wilms tumors.

In the English literature, nuclear positivity has been reported in blastemal and stromal components of Wilms tumor (9,15). In our study, beta-catenin showed cytoplasmic positivity in all stromal, blastemal and epithelial components of WT cases. One case with rhabdoid areas among the cases that had stromal components showed strong immunopositivity with an extent of 80 %. Although nuclear positivity was not detected in our cases, cytoplasmic staining was shown, which means that WNT/beta-catenin pathway might have been activated in Wilms tumors. Besides, cytoplasmic immunopositivity was observed in 7 CCS cases. This signaling pathway has not been studied in CCS cases before.

While beta-catenin was found to be negative in classical mesoblastic nephroma, cytoplasmic positivity was detected in the cellular type.

In contrast to our study, Demellawy et al. indicated that their classical mesoblastic nephroma and mixed mesoblastic nephroma cases showed cytoplasmic staining while the cellular mesoblastic nephroma cases were immunonegative (16).

We showed that 2 renal malignant rhabdoid tumors were weak to moderate immunopositive with a staning percentage of 10-60%. Contrary to our findings, Saito et al. reported immunonegativity in 6 cases of malignant rhabdoid tumors 3 of which were located in kidney (17). In our study, Ewing sarcoma and DLBCL cases were immunonegative.

Our findings suggested that this pathway is activated in WT, CCS, CeMN and rhabdoid tumor. The use of immunohistochemistry in the differential diagnosis is limited.

### PTEN

MicroRNAs (miRNAs) play a role in the development and progression of cancer as an oncogene or tumor suppressor gene. MiR-21 has been reported to show overexpression in almost of all solid tumors studied and play a role in the pathogenesis of renal diseases. MiR-21 regulates multiple target genes, such as PTEN, negatively. PTEN, in particular, suppresses oncogene signaling pathways. In their study on 41 cases of Wilms tumor, Cui et al. have reported a negative correlation between MiR-21 and PTEN levels. Low PTEN protein levels have been shown to correlate with a poor prognosis and late clinical stage (8). Liu et al*.* have performed PTEN immunohistochemistry on 46 WT cases and reported that the tumor did not show strong immunopositivity as much as surrounding normal tissue (7).

In our study, we observed weak immunopositivity in the blastemal and epithelial components of WT cases. In 5 of 16 cases with a stromal component, moderate positivity was found with an extent of 30-100%. Negativity and significant loss of expression was observed in the anaplastic component. In the literature, loss of expression has been generally associated with a poor prognosis in Wilms tumor. According to our findings, negativity and significant loss of expression in the areas of anaplasia might be related with a poor prognosis.

In a study conducted by Little et al., the PTEN mutation was evaluated by the PCR method and only 2 of 12 CCS cases were found to be mutated (18). We observed immunonegativity in all 10 CCS cases. To the best of our knowledge, there is no other study on this subject amongst the documented literature in English. In addition, negativity was detected in our classical mesoblastic nephroma and Ewing sarcoma cases. In order to determine an association with the prognosis, immunohistochemical and molecular studies should be performed in large case series in order to show the association with prognosis.

### PDGFR-Alpha

PDGFR is an angiogenic factor and is encoded by two different genes consisting of A and B chains. The receptor tyrosine kinases, KIT, PDGFR alpha and EGFR, are involved in cell growth and malignant transformation and regulation. Overexpression of PDGFR alpha has been identified in colon, breast, lung, ovarian, and pancreatic carcinomas.

Wetli et al. investigated exon 12,14 and 18 mutations by sequence analysis in 209 Wilms tumor cases, and did not detect the PDGFR alpha mutation; they concluded that PDGFR alpha immunostaining was not reliable (19). Epithelial, stromal, and blastemal components of the 16 WT cases of our study showed immunopositivity with varying intensity and extent. Negativity was found only in the rhabdoid component.

There are no studies in the literature regarding the PDGFR-alpha mechanism in renal tumors except WT. In our study, 9 cases of CCS, 3 CeMN, one rhabdoid tumor, MSFT and DLBCL showed immunpositivity. Ewing sarcoma and classic MN cases were negative.

The use of this immunohistochemical marker in the differential diagnosis is limited, but the role of PDGFR-alpha in the pathogenesis of renal tumors can be investigated.

## CONCLUSION

Immunohistochemically, Cyclin D-1 can be used to differentiate renal clear cell sarcoma from other renal tumors. Loss of expression of PTEN might be associated with a poor prognosis in Wilms tumors. Its role and efficacy in the differential diagnosis with other renal tumors is limited. Although the role of immunohistochemistry is limited, the beta-catenin pathway is used in Wilms tumor, CCS, CeMN, and RT. The use of PDGFR-alpha as an immunohistochemical marker is limited, but its mechanism in renal tumors has not been investigated yet. This can be a subject for future studies.

## Conflict of Interest

The authors declare that they have no conflict of interest.

## Funding

This research received no specific grant from any funding agency in the public, commercial, or not-for-profit sectors.
